# Overexpression of ATAD2 indicates Poor Prognosis in Oral Squamous Cell Carcinoma

**DOI:** 10.7150/ijms.46809

**Published:** 2020-06-27

**Authors:** Xiao-Long Wang, Shuo Wang, Zhi-Zhong Wu, Qi-Chao Yang, Hao Li, Hong-Gang Xiong, Shu-Cheng Wan, Zhi-Jun Sun

**Affiliations:** 1 The State Key Laboratory Breeding Base of Basic Science of Stomatology (Hubei-MOST) & Key Laboratory of Oral Biomedicine Ministry of Education, School & Hospital of Stomatology, Wuhan University, Wuhan, China.; 2 Department of Oral Maxillofacial-Head Neck Oncology, School & Hospital of Stomatology, Wuhan University, Wuhan, China.; 3 Department of Stomatology, Xiangyang Central Hospital, Affiliated Hospital of Hubei University of Arts and Science, Xiangyang, China.

**Keywords:** ATAD2, oral squamous cell carcinoma, prognosis, proliferation, apoptosis, epithelial-mesenchymal transition

## Abstract

ATPase family AAA domain-containing protein 2 (ATAD2) is highly expressed in a variety of malignancies and can promote the proliferation of tumor cells and inhibit their differentiation. However, the expression of ATAD2 and its related mechanism in oral squamous cell carcinoma (OSCC) are still unknown. Immunohistochemical staining of ATAD2, cancer stem cells (CSCs) markers and immune checkpoint molecules was conducted on human OSCC specimens to determine the expression levels of these proteins and their correlations with the clinicopathological characteristics of ATAD2 in OSCC. Moreover, the role of ATAD2 in cell proliferation, apoptosis, migration and epithelial-mesenchymal transition (EMT) were assessed by silencing ATAD2 *in vitro*. Immunohistochemical analysis revealed that ATAD2 expression in OSCC tissues was markedly higher than that in adjacent dysplastic tissues and normal mucosal tissues. Overexpression of ATAD2 was related to poor overall survival in OSCC patients. In addition, the protein expression of ATAD2 was notably correlated with the expression of B7-H4, PD-L1, CMTM6, Slug and ALDH1 in human OSCC. ATAD2 knockdown arrested the cell cycle, promoted the apoptosis, and inhibited the proliferation, migration, and EMT of OSCC cells. In conclusion, these findings revealed that ATAD2 is highly expressed in OSCC and can act as a poor prognostic indicator.

## Introduction

Oral squamous cell carcinoma (OSCC) has a high mortality rate and is the most common head and neck malignancy, accounting for approximately 90% of malignant tumors of the head and neck 1. At present, although surgical excision, chemotherapy, radiotherapy and other comprehensive treatment measures have been taken, the therapeutic effect is still unsatisfactory 2. The focus of OSCC research is to clarify the pathogenesis of OSCC at the molecular level, search for effective screening indicators, further understand relevant molecular mechanisms, and provide new molecular targets for therapy.

ATAD2 (ATPase family AAA domain-containing protein 2), also known as ANCCA (AAA+ nuclear coregulator cancer-associated), is located on human chromosome 8q24.13 3. Studies have shown that ATAD2 is involved in the regulation of chromatin dynamics, transcription and apoptosis, which can facilitate the proliferation of tumor cells and inhibit their differentiation 4. ATAD2 is reported to be highly expressed in multifarious malignant neoplasms, including breast cancer 5, lung cancer 5, gastric carcinoma 6, colorectal cancer 7 and retinoblastoma 8. Overexpression of ATAD2 in tumors often indicates a poor prognosis, and the expression difference is closely associated with tumor size, pathology grade, lymph node metastasis and other clinicopathological factors 9, 10.

EMT plays a vital role in the development of epithelium-derived carcinomas, including OSCC 11. After undergoing EMT, which is related to tumor invasion, metastasis and the development of a cancer stem cell (CSC) phenotype; tumor cells acquire invasive potential, infiltrate into the surrounding matrix, and form a microenvironment that promotes tumor growth and metastasis 12. It has been reported that suppression of EMT is related to the downregulation of ATAD2 in colorectal cancer 13 and renal cell carcinoma 14. These results suggest that ATAD2 may be a biomarker of tumor proliferation and metastasis, as well as a prognostic factor for many human tumors. However, ATAD2 expression in OSCC and its related mechanism are still unknown. In addition, immunotherapy, as a promising novel therapy, has been gradually applied in the clinical treatment of various tumors including OSCC with low toxicity and high specificity 15.The role of ATAD2 in immune cells has not been reported and is worth further study.

The purpose of this study was to investigate the relationship between the expression of ATAD2 in OSCC and its clinicopathological characteristics, the prognostic analysis of OSCC and its biological behavior in OSCC cell lines, and to provide new ideas and a basis for clinical treatment. We detected the expression level of ATAD2 at the tissue level and analyzed its relationship with clinicopathological features. In addition, the correlation of ATAD2 expression with immune checkpoint molecules and CSC specific markers in OSCC was explored. Furthermore, by observing the effect of ATAD2 gene silencing on the proliferation and migration of tumor cells, the relationship between ATAD2 and the occurrence, development and metastasis of oral squamous cell carcinoma was revealed.

## Materials and Methods

### Ethical Statement

The study was performed with the approval of the Institutional Medical Ethics Committee of School and Hospital of Stomatology, Wuhan University (Approval NO.: 2016LUNSHENZI62). Every patient signed a written informed consent form.

### Human OSCC Tissue Microarrays

Tissue samples were obtained from patients diagnosed with OSCC with complete case data after surgical excision at the Department of Oral and Maxillofacial Surgery, School and Hospital of Stomatology, Wuhan University. We classified the patients' OSCC into different clinical stages and determined the histological grades based on the scheme of the World Health Organization guidelines and the Union for International Cancer Control (8th edition) 16. All specimens included 42 oral mucosa specimens adjacent to OSCC, 69 oral epithelial dysplasia specimens, 176 primary OSCC specimens (exclude recurrent, preoperative chemotherapy, or preoperative radiotherapy) 17, 18, 25 recurrent OSCC specimens, 20 OSCC specimens from patients who received preoperative induction TPF (docetaxel, cisplatin, and fluorouracil) chemotherapy, 15 specimens from patients who received preoperative radiotherapy, and 68 metastatic lymph node specimnes in our human OSCC tissue microarrays (T12-412-TMA2, T15-411, T17-790). The method for tissue microarray (TMA) construction was performed as previously described 19.

### Immunohistochemical Staining

Before dewaxing, the tissue microarrays were baked in a 60°C incubator for 60 min. Then the sections were immersed in xylene for 10 min and then soaked in xylene for another 10 min. After this, the slices were dehydrated in gradient ethanol (100%, 95%, 90%, 80%, and 70%) for 5 min each time. Next, antigen retrieval was conducted in citric acid buffer solution (pH 6.0) at 95°C for 20 min and then cooled down at room temperature. Sections were incubated with 3% hydrogen superoxide for 20 min before using normal goat serum at 37°C for 10 min. The primary antibodies against ATAD2 (1:200; Cell Signaling Technology), B7-H4 (1:800; Cell Signaling Technology), PD-L1 (1:100; Cell Signaling Technology), CMTM6 (1:100; Sigma-Aldrich), Slug (1:200; Cell Signaling Technology) and ALDH1 (1:800; Cell Signaling Technology) were incubated in a humidified chamber at 4°C overnight. Subsequently, horseradish peroxidase conjugated goat anti-rabbit secondary antibody was added to the slices for 30 min at 37°C. Then, the sections were counterstained with hematoxylin for approximately 60 sec. TMAs were scanned by an Aperio ScanScope CS scanner (CA, USA) and quantified for pixel quantification by Aperio Quantification software. The immunostaining of the nucleus and membrane was calculated by using the following formula: 1 × (1+) + 2× (2+) + 3× (3+). The expression scores were normalized to a range of 0 to 300. The results of hierarchical clustering analysis were achieved by using Cluster 3.0 with average linkage, and visualization data were obtained using Java TreeView 1.1.3.

### Cell Culture

The OSCC cell lines SCC4, SCC9 and Cal27 were obtained from the American Type Culture Collection (ATCC, Rockville, MD). Tca8113 cells were obtained from the Ninth People's Hospital, Shanghai Jiao Tong University School of Medicine. Cal27 and Tca8113 cells were cultured in Dulbecco's modified Eagle's medium (DMEM) with 4500 mg/L glucose. SCC4 and SCC9 cells were cultured in DMEM/F-12. The two kinds of medium mentioned above were supplemented with 10% fetal bovine serum (FBS, Gibco, USA) and 1% streptomycin-penicillin. All of the OSCC cell lines were cultured in a 37°C incubator and a 5% CO_2_ humidified atmosphere.

### SiRNA and Cell Transfection

The three small interfering RNAs (siRNAs) used for silencing ATAD2 were synthesized by GenePharma (Shanghai, China). Cells were seeded into a 6-well plate 24 hr before cell transfection. We classified the cells into 5 groups: blank (without any transfection), NC (negative control oligonucleotides), siRNA-1 (ATAD2-Homo-507), siRNA-2 (ATAD2-Homo-1121), and siRNA-3 (ATAD2-Homo-2072). Cell transfection was conducted with the Lipofectamine 3000 Transfection Kit (Invitrogen, USA) in accordance with the manufacturer's directions. After transfection for 48 hr at 37°C, the subsequent experiments and analyses were performed.

### Western Blot Analysis

The cells were lysed in cell lysis buffer (Beyotime Biotechnology, China) to obtain the protein, and the concentration of the protein was measured with a BCA Kit (Beyotime Biotechnology, China). Protein (30 μg per lane) was added to a polyacrylamide gel and transferred to a PVDF membrane after separation by electrophoresis. Subsequently, the membrane was blocked with TBST diluted 5% skim milk for 1 hr at room temperature. Primary antibodies against ATAD2 (1:1000; Cell Signaling Technology), E-cadherin (1:1000; Cell Signaling Technology), N-cadherin (1:1000; Cell Signaling Technology) and Snail (1:1000; Cell Signaling Technology) were used for binding protein specifically at 4°C overnight. After washing three times for 10 min in TBST, membranes were incubated with an HRP-labeled goat anti-rabbit IgG (1:10000, Proteintech, Wuhan, China) for 1 hr at 37°C. The WesternBright Sirius Chemiluminescent Detection Kit was used for the visualization of bands on PVDF membranes. GAPDH (1:10000, Bioprimacy) was used as an internal reference protein. The experiments were repeated three times.

### Cell Viability and Colony Formation Assay

Cell suspensions were seeded in 96-well plates at approximately 5000 cells per well. After incubation for 12, 24 and 48 hr, 10 μl of Cell Counting Kit-8 (CCK-8, Dojindo, Japan) solution was added to each well. Cells were cultured in the incubator for 1-4 hr, and the absorbance at 450 nm was measured with a multifunction microplate reader.

Cells in the logarithmic growth phase were harvested and made into single-cell suspensions after 48 hr of transfection. Approximately 1000 cells were seeded into each well of the 6-well plate. When cell clusters were visible to the naked eye, the culture was stopped. Then, the cells were fixed with 4% paraformaldehyde, stained with crystal violet, and counted under a microscope to calculate the clone formation rate.

### Flow Cytometry

After transfection for 48 h, the harvested cells were centrifuged without supernatant and washed with cold PBS 2 times. PBS was aspirated to get the pellet as clean as possible. Next, 1 ml DNA staining solution and 10 microliter permeabilization solution (MULTI SCIENCES, Hangzhou, China) were added to the collected cells. The mixture was mixed by vortex oscillation for 5-10 sec and then incubated in the dark at room temperature for 30 min. The cell cycle was detected by using a flow cytometer (CytoFLEX, BECKMAN COULTER Brea, CA, USA). According to the instructions of an annexin-V-FITC cell apoptosis detection kit (MULTI SCIENCES, Hangzhou, China), 400 μl of annexin V was added to the cells to the cell density was adjusted to 1×10^6^ cells/ml. Five microliters of annexin-V-FITC staining solution was added to the cell suspension, which was mixed gently and incubated at 2-8°C for 15 min in the dark. After staining with propidium iodide (PI) buffer at 4°C in the dark for 5 min, apoptotic cells were detected by using a flow cytometer.

### Wound Healing Assay

After 48 hr of transfection, a 200 μl pipette tip was used to make scratches along the midline of each well when cell confluence reached 90%-100%. Cells were washed three times with sterile PBS to rinse off the detached cells, and then the complete medium was replaced with serum-free medium. After 0, 12 and 24 hr of cell culture, photos were taken. The width of the scratch was recorded to estimate the cell migration ability.

### Transwell Assay

After 48 hr of transfection when cells in the logarithmic growth phase, Cal27 cells were detached and then washed in PBS and serum-free medium successively. Following this, cells were suspended in serum-free medium at an adjusted concentration of 2×10^5^ cells/ml. Then, 800 μl complete medium was added to the bottom of the 24-well plate, and 150 μl cell suspension was added to the upper chamber. After 24 hr of incubation, cells on the upper surface of the membranes were wiped off with cotton swabs. Subsequently, the cells underneath the membranes were fixed in 800 μl of 4% paraformaldehyde for 30 min, and then stained with crystal violet for 20 min. Under an inverted microscope, 5 fields were randomly selected and counted for each group.

### Statistical Analysis

Statistical data analysis was conducted using GraphPad Prism 7 and SPSS 20.0 software. Data between two groups were analyzed using paired/unpaired t-test, and data from multiple groups were analyzed using one-way ANOVA. Kaplan-Meier and log-rank tests were utilized for survival analysis. The best cut-off was confirmed by the website Cutoff Finder 20. The correlation of ATAD2 with other genes was evaluated by two-tailed Pearson's statistics. Data are expressed as the mean ± SEM, and significance was defined as p < 0.05.

## Results

### ATAD2 was overexpressed in human OSCC tissues and significantly linked to overall survival

**Figure 1:**
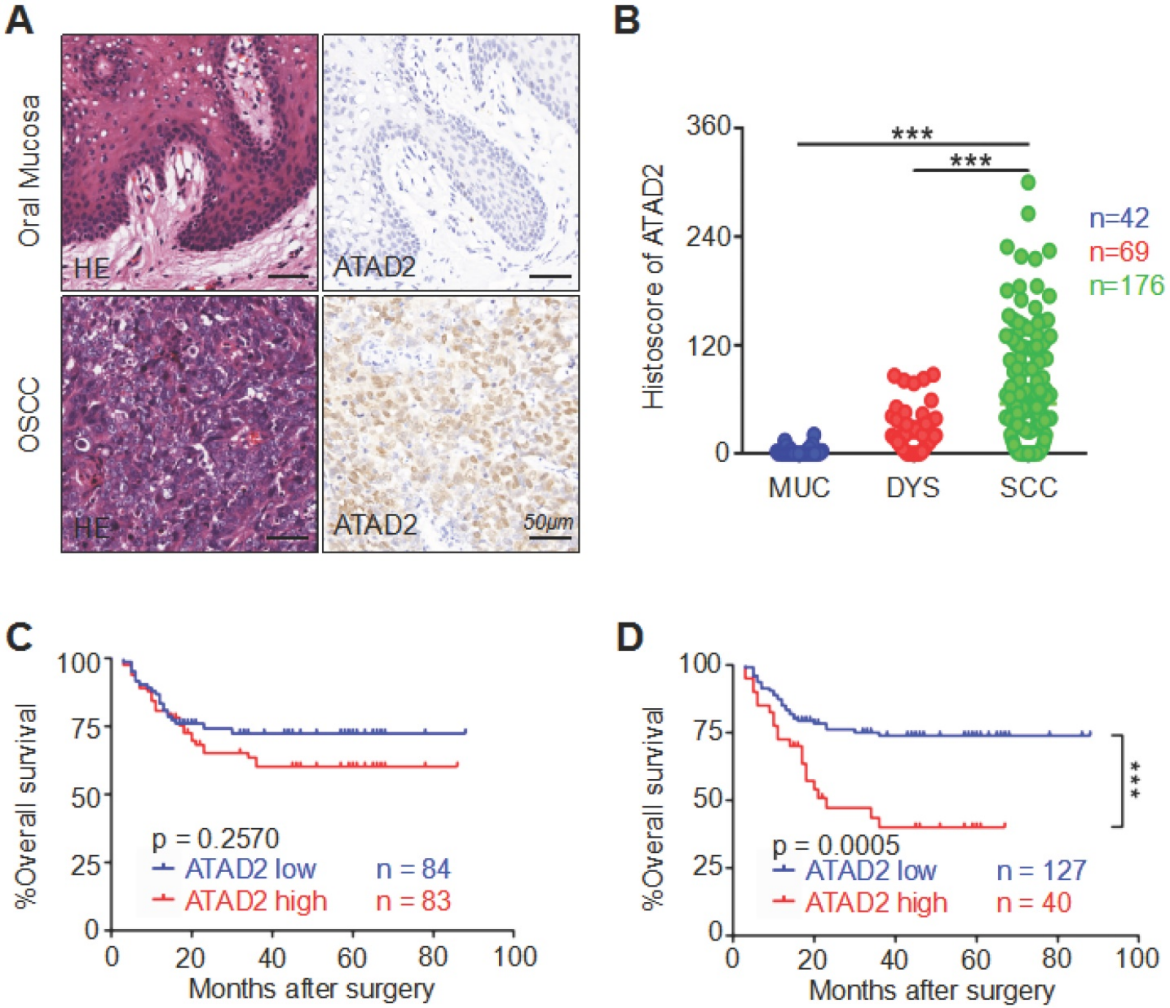
** Overexpression of ATAD2 in primary OSCC.** (**A**) Representative hematoxylin-eosin (HE, left) and immunohistochemical staining (right) of ATAD2 in mucosa adjacent to OSCC and in primary OSCC tissue. Scale bar, 50 µm. (**B**) Quantitative analysis of immunohistochemical staining of ATAD2 in mucosa adjacent to OSCC (MUC, n = 42), epithelial dysplasia (DYS, n = 69) and oral squamous carcinoma (OSCC, n = 176, ***p < 0.001). (**C**) When the median value (histoscore = 20.94946) was used as the cut-off point, the Kaplan-Meier curve shows that the expression of ATAD2 is not related to OSCC prognosis (p=0.2570). (**D**) The Kaplan-Meier curve shows that the high expression of ATAD2 is correlated with the poor prognosis of OSCC when the best cut-off was used (histoscore = 93.7, p = 0.0005).

To detect the correlation between human OSCC and ATAD2 expression, we performed immunohistochemistry on high-throughput tissue microarrays. The immunoreactivity of ATAD2 is characterized by nuclear and cytoplasmic expression in tumor cells, especially in the nucleus, but rarely in the oral mucosa (Figure 1A). Remarkably, the staining intensity of ATAD2 was found to be much stronger in OSCC (n = 176) than in dysplasia (n = 69, p < 0.001) and normal oral mucosa (n = 42, p < 0.001, Figure 1B). Oncomine (www.oncomine.org) database analysis showed that the mRNA expression level of ATAD2 in tougue squamous cell carcinoma (TSCC) was notably higher than that in normal tongue mucosa (p < 0.001, Figure S1A). Moreover, the results from GEPIA (gene expression profiling interactive analysis, gepia.cancer-pku.cn) indicated a significant difference in ATAD2 mRNA expression, with higher expression in head and neck squamous cell carcinoma (HNSCC) than in normal mucosa (p < 0.05, Figure S1B). However, there was no significant difference in expression level between normal mucosa and atypical hyperplasia (p = 0.1646, Figure 1B). Furthermore, the Kaplan-Meier log-rank test was used to evaluate the effect of ATAD2 expression differences on the prognosis of OSCC. As shown in Figure 1C and D, higher ATAD2 expression predicted significantly lower overall survival rates at the best cut-off (p = 0.0005) but not at the median cut-off (p = 0.2570).

### Clinicopathological significance of ATAD2 expression level in human OSCC

**Figure 2:**
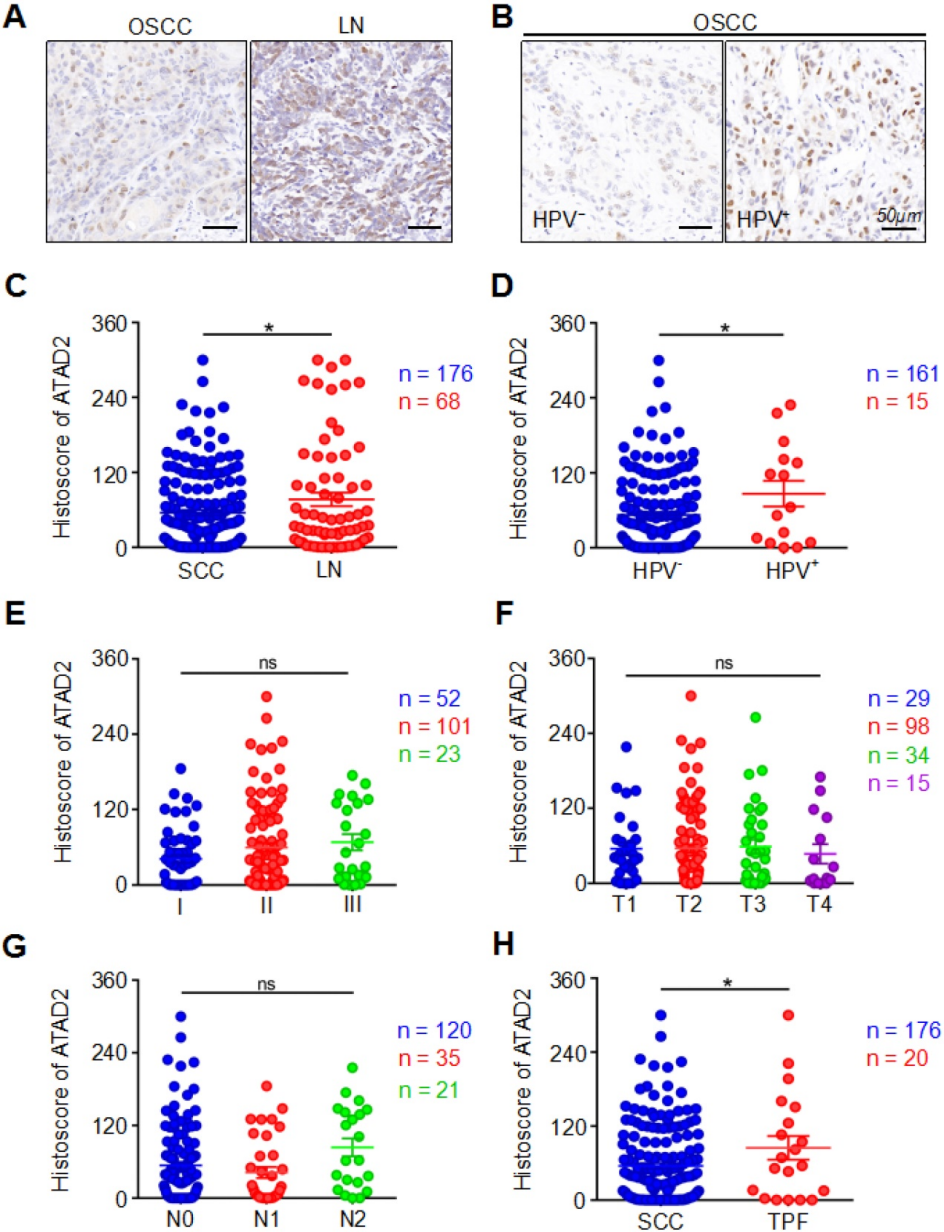
** Clinicopathological significance of ATAD2.** (**A**) Representative immunohistochemical staining of ATAD2 in primary OSCC tissue (SCC, left) and metastatic lymph node (LN, right). Scale bar, 50 µm. (**B**) Immunohistochemical staining of ATAD2 in HPV^-^ (left) and HPV^+^ (right) OSCC tissues. Scale bar, 50 µm. (**C**) Quantitative analysis of the expression level of ATAD2 in primary OSCC and metastatic lymph nodes (OSCC = 176, LN = 68, *p < 0.05). (**D**) Quantitative analysis of ATAD2 expression between HPV^-^ OSCC patients and HPV^+^ OSCC patients (HPV^-^ = 161, HPV^+^ = 15, *p < 0.05). (**E**) Quantitative analysis of the expression level of ATAD2 in different OSCC grades (I = 52, II = 101, III = 23, P > 0.05). (**F**) Quantitative analysis of the expression level of ATAD2 in four tumor sizes (T1 = 29, T2 = 98, T3 = 34, T4 = 15, P > 0.05). (**G**) Quantitative analysis of the expression level of ATAD2 in different lymph node states (N0 = 120, N1 = 35, N2 = 21, P > 0.05). (H) Quantitative analysis of the expression level of ATAD2 in primary OSCC and OSCC after TPF (OSCC = 176, TPF = 20, *P < 0.05).

In our study, the expression of ATAD2 in primary OSCC tissues was markedly lower than that in metastatic lymph nodes (OSCC vs. LN, p = 0.0325, Figure 2A, 2C). Interestingly, we found that the HPV (+) group had markedly higher ATAD2 expression than the HPV (-) negative group (HPV^-^ vs. HPV^+^, p = 0.0416, Figure 2B, 2D). However, neither pathology grade (p > 0.05, Figure 2E), nor tumor size (p > 0.05, Figure 2F) was related to the expression of ATAD2. Moreover, there were no significant differences in ATAD2 expression between the tumors with lymph node metastasis and the tumors without lymph node metastasis (p > 0.05, Figure 2G). Furthermore, ATAD2 expression was correlated with whether presurgical inductive chemotherapy was performed (OSCC vs. TPF, p = 0.0485, Figure 2H). In addition, there was no significant relationship between ATAD2 expression level and tumor recurrence (OSCC vs. recurrence, p > 0.05, Figure S1C). In addition, ATAD2 expression was not significantly different among patients with differences in radiotherapy, drinking or smoking status (OSCC vs. RT, p > 0.05, Figure S1D; drinking vs. nondrinking, p > 0.05, Figure S1E; smoking vs. nonsmoking, p > 0.05, Figure S1F).

### Protein expression of ATAD2 was notably correlated with B7-H4, PD-L1, CMTM6, Slug, and ALDH1 in human OSCC

**Figure 3:**
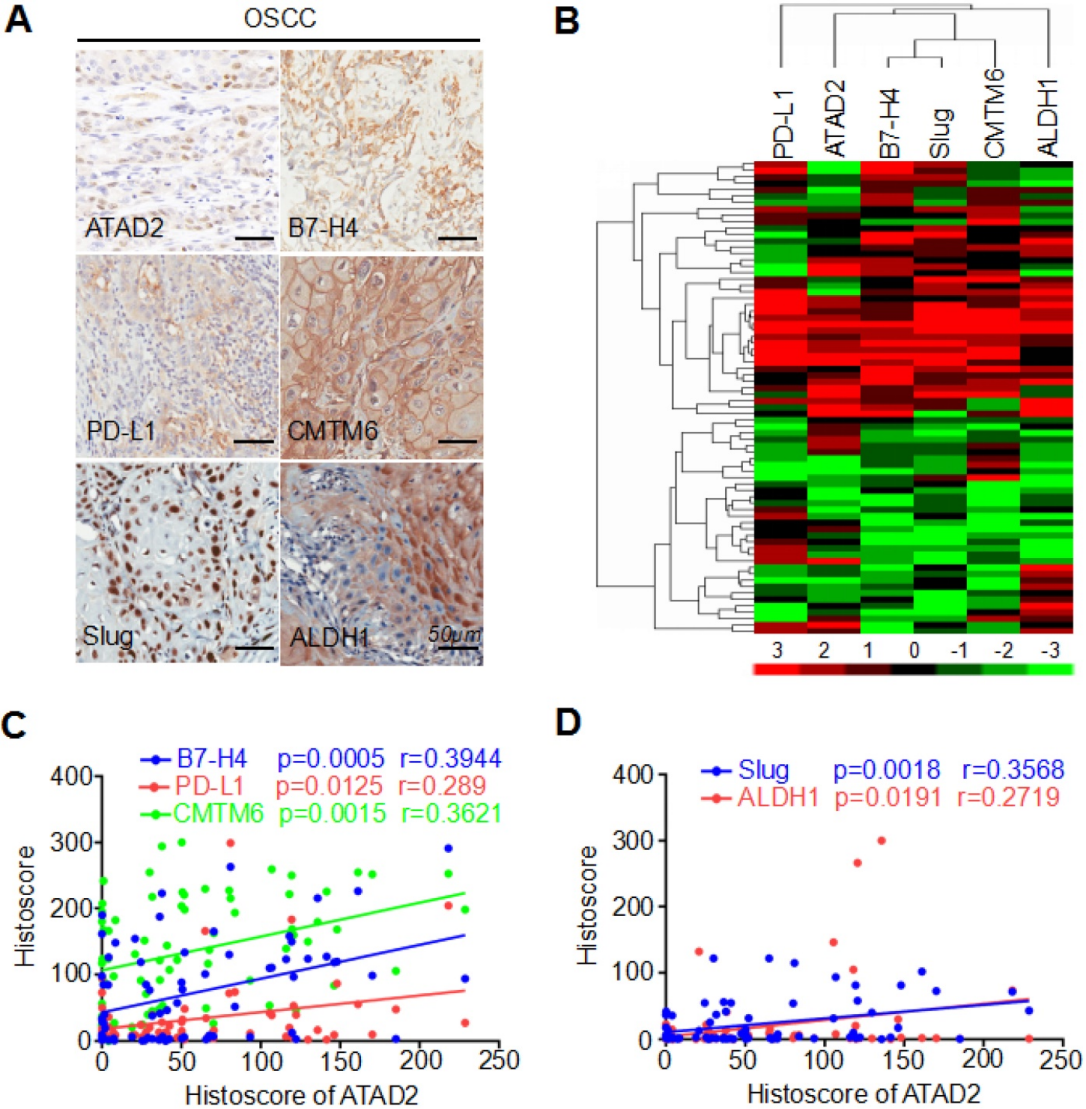
** ATAD2 expression was related to B7-H4, PD-L1, CMTM6, Slug and ALDH1 expression.** (**A**) Reprsentative immunohistochemical staining of ATAD2, B7-H4, PD-L1, CMTM6, Slug and ALDH1 in OSCC. Scale bar, 50 µm. (**B**) Hierarchical clustering depicts the correlation of ATAD2, B7-H4, PD-L1, CMTM6, Slug and ALDH1 in OSCC. (**C**) Pearson's correlation coefficient test of ATAD2 with B7-H4 (p = 0.0005, r = 0.3944), PD-L1 (p = 0.0125, r = 0.289) and CMTM6 (p = 0.0015, r = 0.3621). (**D**) Pearson's correlation coefficient test of ATAD2 with Slug (p = 0.0018, r = 0.3568) and ALDH1 (p = 0.0191, r = 0.2719).

We applied immunohistochemistry to human OSCC TMAs and found high protein expression levels of B7-H4, PD-L1, CMTM6, Slug and ALDH1 (Figure 3A). Next, hierarchical clustering analysis showed that ATAD2, B7-H4, PD-L1, CMTM6, Slug and ALDH1 had similar trends in terms of protein expression levels (Figure 3B). Additionally, Pearson's correlation analysis indicated that the expression level of ATAD2 was statistically correlated with B7-H4 (p = 0.0005, r = 0.3944), PD-L1 (p = 0.0125, r = 0.289), CMTM6 (p = 0.0015, r = 0.3621), Slug (p = 0.0018, r = 0.3568) and ALDH1 (p = 0.0191, r = 0.2719), as shown in Figure 3C and D.

### Silencing of ATAD2 reduced OSCC cell proliferation

**Figure 4:**
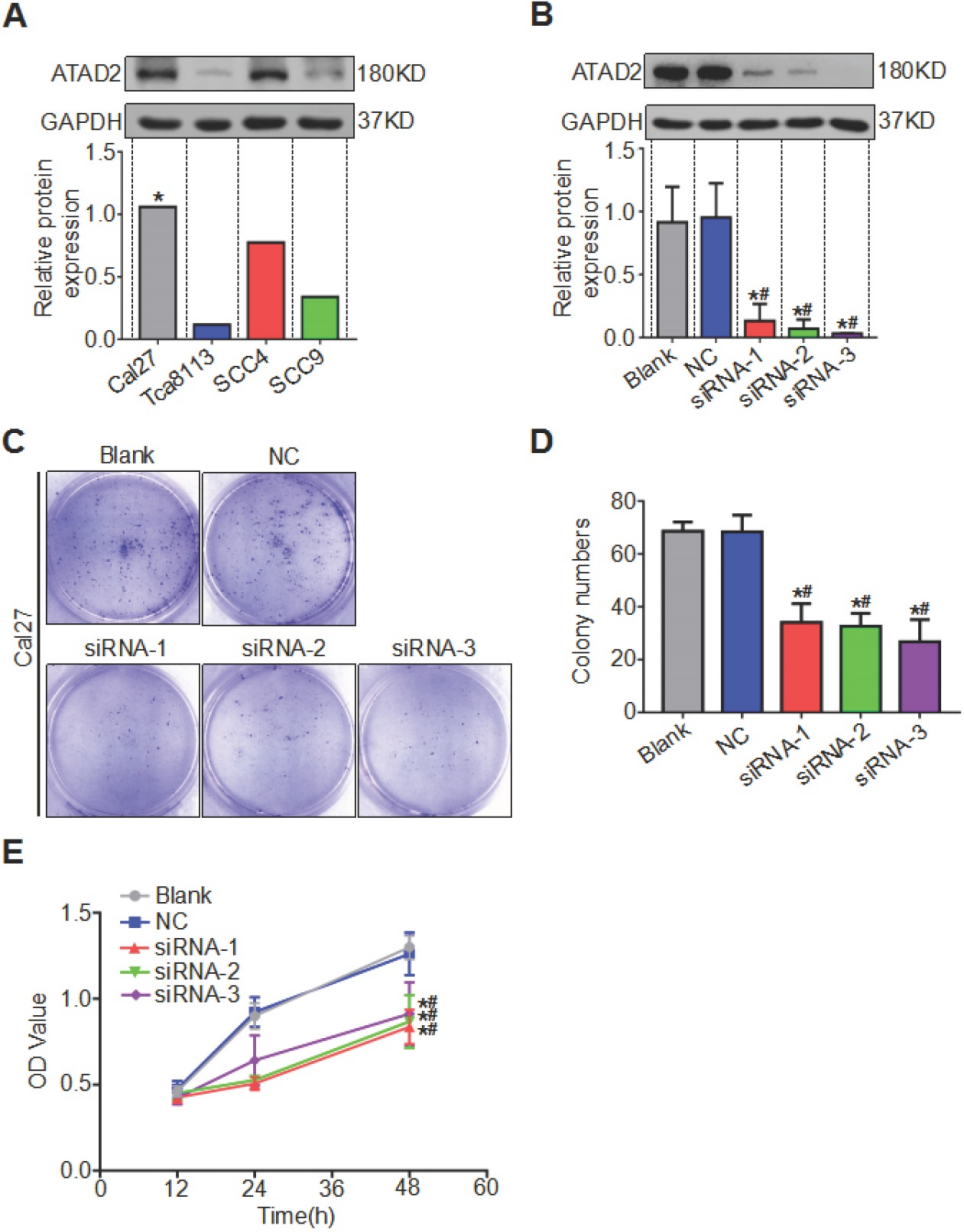
** Silencing of ATAD2 decreased OSCC cell proliferation.** (**A**) Western blot analysis was performed to examine ATAD2 expression in several OSCC cell lines (*, the relative highest expression level). (**B**) Western blot analysis was performed to examine ATAD2 expression after ATAD2 siRNA transfection in Cal27 cell lines. (**C**) Representative images of anchor-dependent colony formation assays in Cal-27 cells. (**D**) Quantitative analysis of anchor-dependent colony formation assays in Cal27 cells. (**E**) Growth curves of ATAD2 knockdown groups or blank and NC groups as measured by a CCK-8 assay (*P < 0.05 vs. the blank group, ^#^P < 0.05 vs. the NC group).

We compared the expression levels of ATAD2 in OSCC cell lines Tca8113, Cal27, SCC4 and SCC9 by western blotting analysis and discovered that the protein content of ATAD2 in Cal27 was relatively the highest, so we chose the Cal27 cell line for subsequent experiments (Figure 4A). All three siRNA sequences (ATAD2-Homo-507, ATAD2-Homo-1121 and ATAD2-Homo-2072) used to knockdown ATAD2 in Cal27 cell lines had high knockout efficiency (Figure 4B). We conducted a colony formation assay to observe the influence of ATAD2 knockdown on OSCC cell proliferation. As shown in Figure 4 C and D, the colony numbers of the siRNA groups were markedly decreased compared with those of the blank and NC groups. In addition, the CCK-8 assay was applied to detect cell proliferation at 12, 24, and 48 hr. Compared with that in the blank group and the NC group, the OD value of siRNA groups was gradually augmented from 12 to 48 hr ( Figure 4E).

### ATAD2 knockdown promoted OSCC cell apoptosis and weakened cell cycle progression

**Figure 5:**
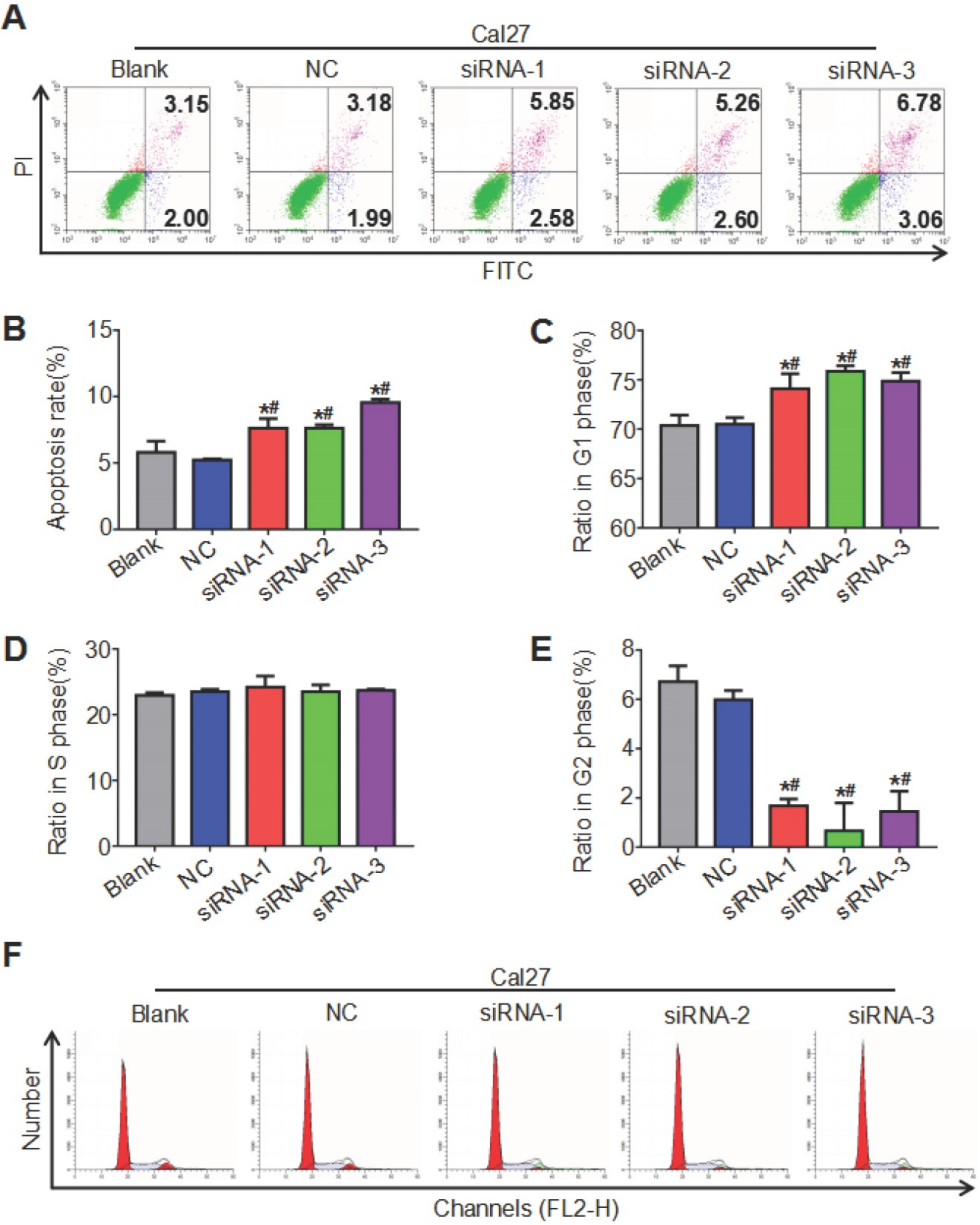
** ATAD2 downregulation enhanced OSCC cell apoptosis and attenuated OSCC cell cycle progression.** (**A**) Flow cytometry plot demonstrating cell apoptosis status. (**B**) Quantitative analysis of the apoptosis rate of Cal27 cells in response to treatment with siRNA against ATAD2. (**C**) Quantitative analysis of the ratio of Cal27 cells in G1 phase in response to treatment with siRNA against ATAD2. (**D**) Quantitative analysis of the ratio of Cal27 cells in S phase in response to treatment with siRNA against ATAD2. (**E**) Quantitative analysis of the ratio of Cal27 cells in G2 phase in response to treatment with siRNA against ATAD2. (**F**) Flow cytometry plot demonstrating cell cycle distribution (*P < 0.05 vs. the blank group, ^#^P < 0.05 vs. the NC group).

Both the cell cycle and apoptosis were detected by flow cytometry, and the cell cycle was analyzed by ModFit, while apoptosis was analyzed by CytExpert. The results showed that the cell apoptosis rate of the siRNA groups increased compared with that of the blank group and the NC group, and the difference between the blank group and the NC group was not significant (Figure 5A, 5B). As seen in Figure 5 C, D, E and F, the siRNA groups had prolonged G1 phase (more cells) and shortened G2 phase (fewer cells) relative to the blank group and NC group, while the proportion of cells in S phase of different groups was not significantly different. These findings indicated that downregulation of ATAD2 can induce the apoptosis of OSCC cells and block the cell cycle in G1 phase *in vitro*.

### Knockdown of ATAD2 suppressed OSCC cell migration and affected the expression of EMT biomarkers in OSCC

**Figure 6:**
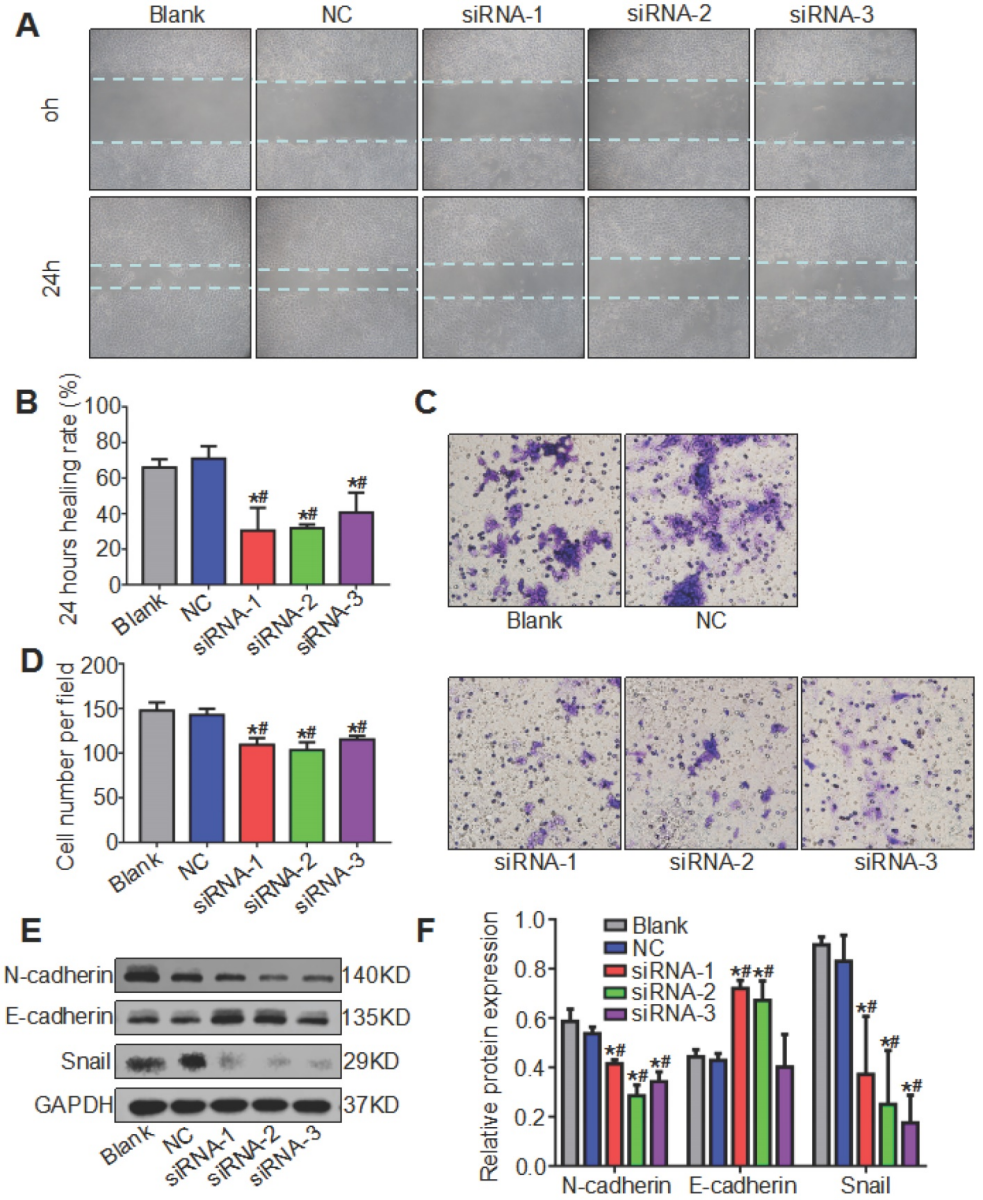
** Knockdown of ATAD2 inhibited cell migration and altered the expression of EMT biomarkers.** (**A**) Representative photographs of the wound healing assay (magnification, 10×). Photographs were taken at 0 and 24 hr after wounding. (**B**) Quantitative statistics of the healing rate. (**C**) Representative microphotographs of the Transwell assay (magnification, 20×). Photographs were taken at 24 hr after cell plating. (**D**) Quantitative statistics of migrated cells. (**E**) Western blot results showed a decrease in mesenchymal-related proteins N-cadherin and Snail and an increase in epithelial-related protein E-cadherin after ATAD2 siRNA transfection in Cal27 cell lines. (**F**) Quantitative analysis of the protein expression levels of ATAD2 and EMT biomarkers (*P < 0.05 vs. the blank group, ^#^P < 0.05 vs. the NC group).

We mentioned earlier that ATAD2 was more highly expressed in metastatic lymph nodes than in OSCC tissues, so we hypothesized that ATAD2 may be associated with the migration of OSCC cells. To evaluate the effect of ATAD2 on cell migration, wound healing and Transwell assays were conducted. As shown in Figure 6 A and B, the 24 hr healing rate was markedly reduced in the siRNA groups compared with the blank group and NC group. In the Transwell assay, more migrating cells that passed through the filter were observed in the blank group and NC group compared with the siRNA groups (Figure 6C, 6D). Moreover, we extracted the total protein of OSCC cells after transfection and detected whether the increased cell migration ability was related to EMT by western blotting. As shown in Figure 6E, when ATAD2 was knocked out, the expression levels of Snail and N-cadherin decreased, while E-cadherin increased. Taken together, the results indicated that silencing ATAD2 inhibited OSCC migration and EMT progression.

## Discussion

The discovery of the ATAD2 gene is a new breakthrough in the field of tumor molecular biology. Studies have shown that ATAD2 is an oncogene involved in the development of various tumors 21. Our findings revealed that overexpression of ATAD2 predicted a poor prognosis in patients with OSCC. Attenuation of ATAD2 interfered with the cell cycle, promoted the apoptosis, and inhibited the proliferation, migration, and EMT of OSCC cells.

In this study, immunohistochemical analysis showed that the expression of ATAD2 in human OSCC tissues was significantly higher than that in adjacent dysplastic tissues and normal mucosal tissues. In addition, the study revealed that overexpression of ATAD2 was related to poor overall survival in OSCC patients. Upregulaton of ATAD2 in cancer tissues compared to paired normal tissues has been reported previously in multiple cancers, such as renal cell carcinoma 22, breast cancer 23 and prostate cancer 24. Hwang et al. reported that ATAD2 expression unfavorably affected disease-specific survival (DDS) in hepatocellular carcinoma, and the 5-year DSS rate of the ATAD2-positive group was notably lower than that of the ATAD2-negative group 9. Zheng et al. reported that compared with that of cervical cancer patients with low ATAD2 expression, the overall survival of patients with high ATAD2 expression was significantly reduced 25. To the best of our knowledge, this is the first study to explore the expression of ATAD2 in human OSCC and to assess the clinicopathological and prognostic value of ATAD2. The findings above suggest that ATAD2 might be not only a biomarker for diagnosis but also a prognostic indicator in OSCC. However, mucosa adjacent to OSCC could not represent normal mucosa due to the concept of field carcinogenesis 26. The lack of independent healthy samples not related to studied tumors limited the diagnostic value of the biomarker.

Studies have shown that ATAD2 is closely associated with various regulatory mechanisms in tumor cells, including cell proliferation and tumor metastasis 10, 27, 28. Apoptosis plays a vital role in the clearance of diseased cells. It is one of the key factors of tumor occurrence and development that lead to abnormal apoptosis and even cause cells to lose the ability of apoptosis. Cell cycle entry was blocked by ATAD2 downregulation in retinoblastoma 8. Our findings showed that through small RNA interference targeting ATAD2, tumor cell proliferation was inhibited and apoptosis and G1 cell cycle arrest were induced.

In the tumorigenesis of malignant tumors, including OSCC, EMT plays an extremely important role, which changes cell morphology, reduces cell polarity and increases cell motility 29, 30. E-cadherin is an important adhesion molecule that maintains the connections between epithelial cells and maintains the epithelial phenotype. The reduction or loss of E-cadherin is a key factor in the occurrence of EMT 31. A previous study showed that silencing ATAD2 inhibited the migration and invasion of colorectal cancer cells by suppressing EMT 13. Similar studies indicated that the inhibition of EMT in renal cell carcinoma cells is regulated by ATAD2 14. Herein, our evidence suggests that low ATAD2 expression is related to reduce EMT in OSCC cells and further affects tumor metastasis. In addition, we found that the expression of ATAD2 was related to the expression of Slug, ALDH1 and CMTM6. Slug, a zinc finger transcriptional suppressor, is an EMT marker 32. Previous studies indicated that EMT progression is a pivotal regulator of the CSC phenotype 33. In addition, the silencing of Slug results in downregulation of ALDH1 expression and repression of CSC properties, which suggests that EMT participates in the origination of CSCs 34. Research has found that CMTM6 knockdown inhibits stem cell-like properties and TGF-β-induced EMT to varying degrees 35. These results suggest that ATAD2 may play a vital role in the occurrence and development of tumors by regulating EMT and the CSC-like phenotype.

B7-H4 and PD-L1 (PD-1 ligand), as inhibitory immune checkpoints, are often exploited by cancer cells to evade immunosurveillance in OSCC 36 and have been proven to be prognostic biomarkers in OSCC 37, 38. In addition, CMTM6 has been proven to have the capacity to maintain the expression of PD-L1 and regulate antitumor immunity 39. Our study found that ATAD2 was markedly correlated with B7-H4, PD-L1 and CMTM6 in OSCC. Therefore, these findings indicate that ATAD2 may contribute to immune repression in the tumor microenvironment, and further demonstrate that ATAD2 may be a poor prognostic indicator.

In conclusion, the current research demonstrated that ATAD2 is highly expressed in OSCC and may act as a poor prognostic indicator. Moreover, the correlation with B7-H4, PD-L1, Slug, ALDH1 and CMTM6 may predict that ATAD2 plays a potential role in EMT, immunosuppression and regulation of CSCs in OSCC. Furthermore, silencing ATAD2 inhibits OSCC cell proliferation, migration and EMT and enhances cell apoptosis. These findings indicate that ATAD2 may be a logical oncogene candidate and a target for the treatment of OSCC. However, in our study, there were still some limits of prognostic data deriving from variable cut-off selection, due to short follow up period and low-numbered studied population. Furthermore, it has not been confirmed whether the *in vivo* effects of ATAD2 are similar to the *in vitro* effects; animal studies have not been conducted, and further study on the role of ATAD2 in animals is needed.

## Supplementary Material

Supplementary figure.
