# Overexpression of ATAD2 indicates Poor Prognosis in Oral Squamous Cell Carcinoma: Erratum

**DOI:** 10.7150/ijms.112159

**Published:** 2025-03-28

**Authors:** Xiao-Long Wang, Shuo Wang, Zhi-Zhong Wu, Qi-Chao Yang, Hao Li, Hong-Gang Xiong, Shu-Cheng Wan, Zhi-Jun Sun

**Affiliations:** 1 The State Key Laboratory Breeding Base of Basic Science of Stomatology (Hubei-MOST) & Key Laboratory of Oral Biomedicine Ministry of Education, School & Hospital of Stomatology, Wuhan University, Wuhan, China.; 2 Department of Oral Maxillofacial-Head Neck Oncology, School & Hospital of Stomatology, Wuhan University, Wuhan, China.; 3 Department of Stomatology, Xiangyang Central Hospital, Affiliated Hospital of Hubei University of Arts and Science, Xiangyang, China.

When reviewing the previous work, we realized the Figure 3A in the published article has mistakenly used images. Specifically, the representative IHC image of “Slug” in Figure 3A was misused. The corrected Figure 3A is displayed below. We sincerely apologize for this error. All authors have confirmed that the mistake does not affect the original conclusions.

## Figures and Tables

**Figure 3A:**
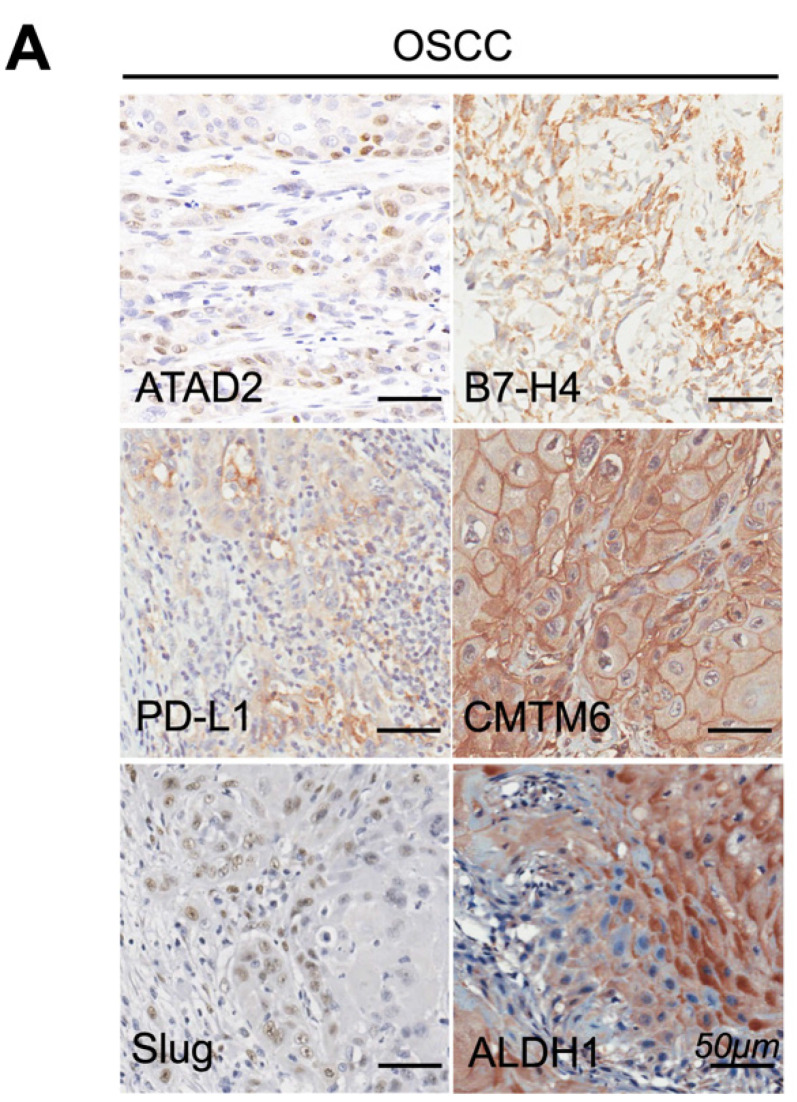
Representative immunohistochemical staining of ATAD2, B7-H4, PD-L1, CMTM6, Slug and ALDH1 in OSCC. Scale bar, 50 µm.

