# Supplementary figures and images for: Overexpression of ATAD2 indicates Poor Prognosis in Oral Squamous Cell Carcinoma
Source: Int J Med Sci. 2020 Jun 27;17(11):1598–609. doi: 10.7150/ijms.46809 (PMC7359390; doi:10.7150/ijms.46809)

# Supplementary Figure

**A**

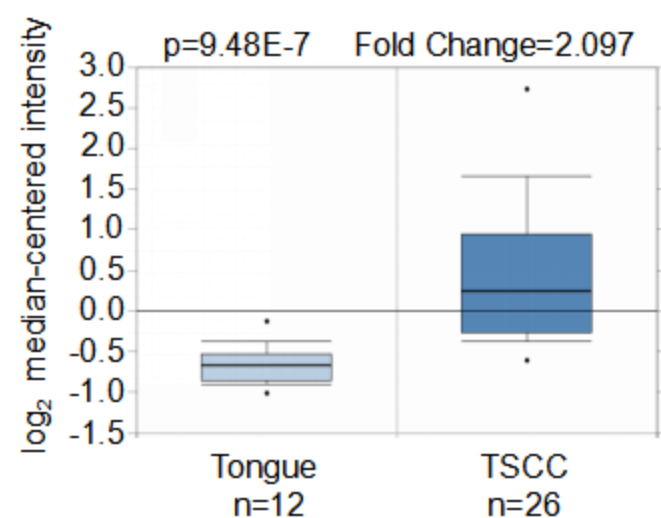

**B**

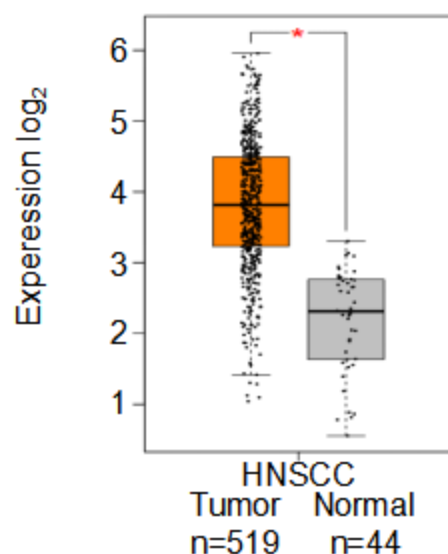

**C**

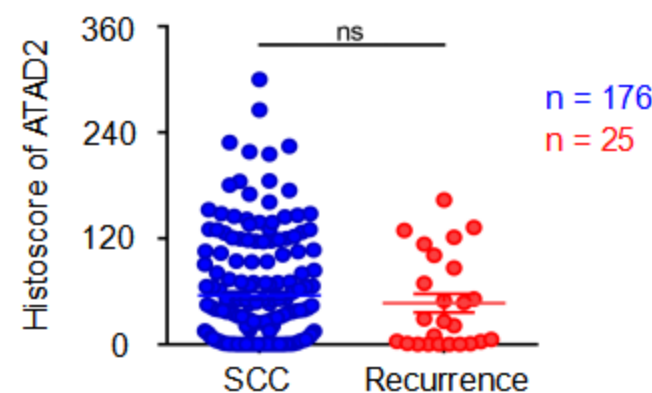

**D**

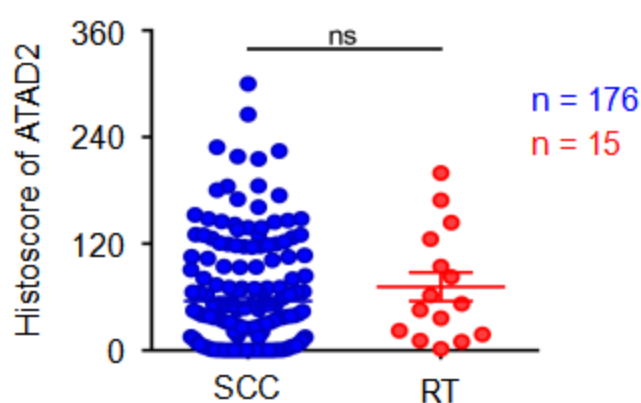

**E**

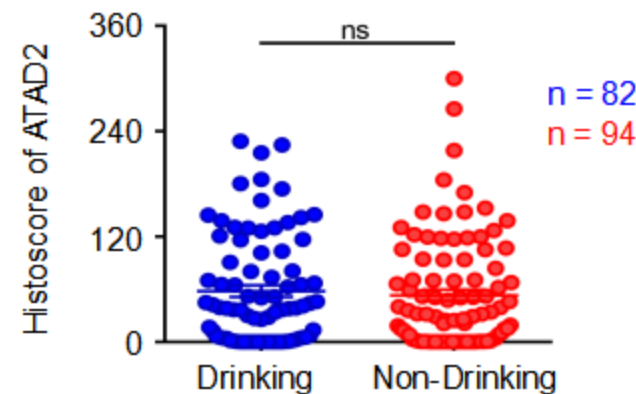

**F**

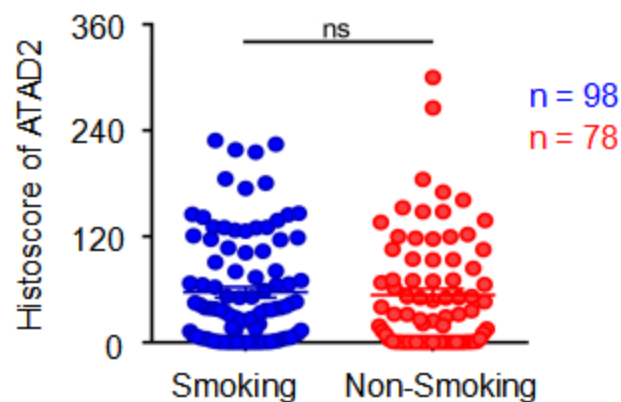

Supplement: Supplementary file 1 — Supplementary figure. [file ijmsv17p1598s1.pdf]
